# Angioregulatory microRNAs in Colorectal Cancer

**DOI:** 10.3390/cancers12010071

**Published:** 2019-12-26

**Authors:** Mohammad Hasan Soheilifar, Michael Grusch, Hoda Keshmiri Neghab, Razieh Amini, Hamid Maadi, Massoud Saidijam, Zhixiang Wang

**Affiliations:** 1 Research Center for Molecular Medicine and Genetics, School of Medicine, Hamadan University of Medical Sciences, Hamadan 65178, Iran; soheilih@gmail.com (M.H.S.); sjam110@yahoo.com (M.S.); 2 Department of Medicine I, Institute of Cancer Research, Medical University of Vienna, 1090 Vienna, Austria; michael.grusch@meduniwien.ac.at; 3 Department of Photo Healing and Regeneration, Medical Laser Research Center, Yara Institute Academic Center for Education, Culture and Research (ACECR), Tehran 1315795613, Iran; hodakeshmiri@ut.ac.ir; 4 Department of Medical Genetics, and Signal Transduction Research Group, Faculty of Medicine and Dentistry, University of Alberta, Edmonton, AB T6G 2H7, Canada; hmaadi@ualberta.ca

**Keywords:** colorectal cancer 1, angiogenesis 2, microRNA 3

## Abstract

Colorectal cancer (CRC) is one of the leading causes of cancer mortality. Angiogenesis is a rate-determining step in CRC development and metastasis. The balance of angiogenic and antiangiogenic factors is crucial in this process. Angiogenesis-related genes can be regulated post-transcriptionally by microRNAs (miRNAs) and some miRNAs have been shown to shuttle between tumor cells and the tumor microenvironment (TME). MiRNAs have context-dependent actions and can promote or suppress angiogenesis dependent on the type of cancer. On the one hand, miRNAs downregulate anti-angiogenic targets and lead to angiogenesis induction. Tumor suppressor miRNAs, on the other hand, enhance anti-angiogenic response by targeting pro-angiogenic factors. Understanding the interaction between these miRNAs and their target mRNAs will help to unravel molecular mechanisms involved in CRC progression. The aim of this article is to review the current literature on angioregulatory miRNAs in CRC.

## 1. Introduction

Angiogenesis is a coordinated multi-step process, which serves to fulfill nutrient and oxygen demand of normal and cancerous tissues and can be controlled by microRNAs (miRNAs) at multiple levels. MiRNAs are small non-coding RNAs comprised of 18–25 bases and they post-transcriptionally regulate gene expression including the expression of oncogenes and tumor suppressor genes in cancer [1,2,3]. MiRNAs bind to the 3′-untranslated regions (3’-UTRs) of target mRNAs and repress translation or cause transcript degradation. Functional studies by miRNA overexpression or inhibition have revealed prominent roles of miRNAs in various types of cancer including colorectal cancer (CRC). In addition to their pivotal role in tumor cell migration and invasion, miRNAs can modulate the expression of angiogenic or anti-angiogenic factors. Imbalance between these factors leads to dysregulation of angiogenesis and aberrant vascular architecture in cancer [4]. Endothelial cells (ECs) are a major component of the (tumor microenvironment) TME and formation of neo-vessels is critical in CRC growth and metastasis [5]. The metastatic and angiogenic potential of CRC cells depends on interaction with ECs through various signals including the transfer of molecules via exosomes. Exosomes are a type of extracellular vesicles that have a size of 30–100 nm and contain growth factors, lipids, and non-coding RNAs, which are involved in the communication between tumor and stroma cells [6]. Exosomes are taken up by target cells through different pathways such as cell membrane fusion and receptor-mediated endocytosis [7]. Exosomal angio-miRNAs and other angiogenesis-related factors released from CRC cells can transport angiogenic signals to ECs. Multiple signaling molecules and pathways such as hypoxia-inducible factor 1*α* (HIF1α), vascular endothelial growth factor (VEGF)/vascular endothelial growth factor receptor (VEGFR), phosphoinositide 3-kinases (PI3K)/protein kinase B (AKT)/mammalian target of rapamycin (mTOR), transforming growth factor β (TGFβ), extracellular-signal-regulated kinase (ERK) and WNT/β-catenin contribute to CRC angiogenesis. Interestingly, miRNAs are crucial regulators of these factors and pathways. Moreover, anti-angiogenic therapies have improved the survival of CRC patients. Therefore, investigation of angiogenesis-related miRNAs could help to find effective non-coding RNA-based drugs and novel diagnostic, prognostic, or predictive biomarkers. For example, anti-miR-21 could bind to pri-miR-30 and inhibit tubulogenesis in CRC [8]. In this review, we discuss the current state of research on the roles of angioregulatory miRNAs in CRC. 

## 2. MiRNAs Regulate Physiological and Pathological Angiogenesis

Angiogenesis normally occurs during physiological processes like embryonic development, wound healing, and the menstrual cycle. Embryonic stem cell differentiation to endothelial progenitor cells and ECs is regulated by angio-miRs during embryonic development [9] and also placenta angiogenesis in pregnancy can be modulated by miRNAs [10]. MiR-17, -20 and -20b contribute to placenta angiogenesis through targeting *EPHB4* and *ephrin-B2* and their differential expression in preeclampsia compared with normal pregnancies suggests angioregulatory roles of these miRNAs in placenta pathogenesis [11]. It has been reported that recurrent miscarriage is linked to aberrant expression of miR-16 in villi and decidua in addition to peripheral mononuclear cells [12,13]. Neoangiogenesis is a critical step in wound healing by providing nutrient and oxygen supply at the wound site. Angioregulatory functions of miR-148b, miR-615-5p, miR-200b, miR-27b, miR-21, and miR-199a-5p in wound healing have been investigated in several studies (Table 1). 

MiRNAs also regulate endometrium function and differentiation into the receptive state in the menstrual cycle [14]. Extracellular miRNAs can be involved in endometrial-peritoneal interactions which modulate angiogenesis in endometriosis. An in vitro study demonstrated that peritoneal fluid treatment of endometrial cell cultures resulted in decreased expression of miR-16, -17-5p, -20a, -125a, -221, and -222 which led to increased *VEGFA* expression [15].

## 3. CRC Progression and Metastasis Are Regulated by MiRNA-Mediated Crosstalk between Tumor Cells and the TME 

**Figure 1:**
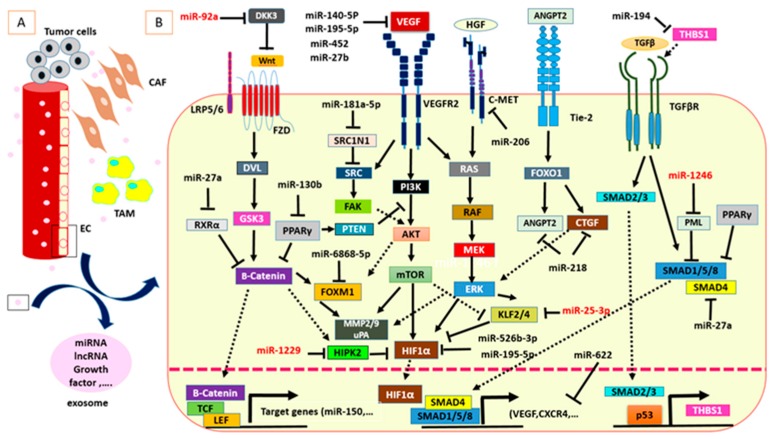
MiRNAs are critical mediators in CRC angiogenesis. (**A**) Communication between CRC cells and TME components such as ECs could be mediated by exosomes. (**B**) Intracellular and exosome-derived miRNAs are involved in important molecular pathways of CRC angiogenesis. Exosome-derived miRNAs are shown in red color.

Endothelial cells, fibroblasts, tumor-associated macrophages (TAMs), pericytes, and lymphocytes contribute to tumorigenesis through various activities such as angiogenesis dysregulation, immune evasion, growth factor overexpression, and extracellular matrix modification. Cancer-associated fibroblasts (CAFs) are major players in the TME and contribute to tumor-stroma interactions. Bhome et al. have shown miR-329, miR-181a, miR-199b, miR-382, miR-215, and miR-21 to be enriched in CAF-derived exosomes in CRC [27]; Moreover, they confirmed miR-21 transfer from CAFs to CRC cells which led to increased tumor cell invasion and metastasis. TAMs are another crucial component of the tumor stroma. These cells can have a dual effect on tumorigenesis [28]. M2 macrophage- derived exosomes containing miR-21-5p and miR-155-5p were shown to target brahma-related gene 1 (*BRG1*) when transmitted to CRC cells and thereby inhibited metastasis [29]. Oncogenic role of *BRG1* through Wnt3a upregulation have been demonstrated in CRC both in vitro and in vivo [30]. Increased expression of BRG1 is correlated with epithelial–mesenchymal transition (EMT) marker SNAI and associated with poor prognosis in CRC patients [31]. TAM also regulate STAT3-mediated suppression of miR-506-3p in CRC [32]. Another miRNA which is involved in CRC and TME interplay is miR-506-3p. *FoxQ1* inhibition via miR-506-3p and subsequent CCL2 upregulation can promote circulating tumor cell (CTC)-mediated tumor metastasis in CRC patients [32]. The angiogenic switch of CRC involves VEGF secretion from cancer cells under hypoxic conditions which triggers angiogenesis via VEGFR expressed on ECs. MiRNAs were shown to suppress *VEGF* expression in tumor cells and *VEGFR* expression in ECs [33,34]. Thus, on the one hand, miRNAs can regulate communication between tumor cells and different components of the TME by modulating the expression of growth factors or their receptors. Angioregulatory miRNAs can be transferred to various cells in the tumor niche via exosomes and shuttling of miRNAs between tumor cells and cells of the TME is an important aspect in cellular communication (Figure 1). Importantly, exosomal miRNAs can be detected in body fluids and serve as non-invasive biomarkers in CRC [35]. Several putative angio-miRs were investigated in CRC via anti-miRNA oligonucleotides or overexpression of miRNAs and the respective studies will be discussed below (Table 2 and Table 3).

## 4. Pro-Angiogenic MiRNAs in CRC

MiR-92a: MiR-92a is a member of the miR-17-92 cluster which is a prominent oncomiR cluster in CRC. MiR-17~92 promotes CRC angiogenesis via targeting transforming growth factor β type II receptor (*TGFβR2*), *HIF1α*, and *VEGFA* [101]. A miR-92a/KLF4/p21 axis facilitates CRC cell proliferation and migration [102]. Exosome-mediated transfer of miR-92a from colon cancer cells to endothelial cells leads to angiogenesis induction through downregulation of Dickkopf-3 (*DKK3*) and claudin-11 [36]. Several studies have shown that DKK3 has a diverse function in tumor angiogenesis and oncogenesis [103,104,105,106]. Busceti et al. have indicated an angio-promoting role of DKK3 via VEGF upregulation [107]. DKK3 upregulation in CRC tissue compared to normal adjacent tissue correlated with increased microvessel formation [108]. DKK3 interaction with β2-microglobulin (β2M) inhibits VEGFR-2/Akt/mTOR signaling activation in ovarian cancer and inhibits angiogenesis [109]. DKK3 also modulates the Wnt/β-catenin signaling pathway and could be a diagnostic and prognostic biomarker in the serum of CRC patients [110]. Claudin-11 belongs to the claudin transmembrane protein family which is required for the formation of endothelial cell tight junctions. Tight junctions are involved in extravasation and angiogenesis [111]. Claudin-11 hyper-methylation is linked to colon cancer progression and metastasis [112].

MiR-1246: miR-1246 is a highly expressed oncomiR in CRC tissue and serum compared to controls and was identified as a novel CRC biomarker [113,114]. MiR-1246 enhances tumor growth, invasion, and metastasis and is involved in chemoresistance and self-renewal ability, a characteristic feature of cancer stem cells in CRC [115,116]. Pri-miR-1246 methylation by METTL3 (methyltransferase) upregulation can facilitate miR-1246 maturation and subsequently MAPK signaling activation in CRC [117]. Exosome-derived miR-1246 can be taken up by HUVECs and promotes angiogenesis through promyelocytic leukemia protein (*PML*) inhibition and SMAD 1/5/8 signaling activation [37]. A number of reports have demonstrated a significant role of SMAD signaling in angiogenesis [118,119]. TGF-β/SMAD mediated angiogenesis could be further enhanced by cooperation with insulin in EC [120]. PML could exert its antiangiogenic roles in IFN-α-dependent manner. In addition, PML negatively regulates angiogenesis via suppression of mTOR-HIF1α [121]. HIF-1α is a key player in hypoxic signaling and tumor neovascularization. Sorafenib is a multi-targeted tyrosine kinase inhibitor which inhibits CRC angiogenesis and proliferation [122]. A panel of miRNAs including miR-1246 were shown to have decreased expression in response to Sorafenib treatment in the Caco-2 cell line [123].

MiR-1229: miR-1229 is part of a panel of 5 serum miRNAs that provides superior specificity and sensitivity for early detection of CRC and could be applied to distinguish CRC from colorectal adenomas and healthy individuals [124]. CRC-derived exosomal miR-1229 is elevated in the serum of patients and leads to tube formation in HUVECs in vitro through *HIPK2* inhibition and subsequent VEGF upregulation [38]. An inverse association between HIPK2 and VEGF expression supports an antiangiogenic activity of HIPK2 [125]. Moreover, HIPK2 activates p53 and inhibits tumorigenesis [126]. 

MiR-25-3p: MiR-25 has a contradictory role in CRC development and its tumor suppressive or oncogenic function in CRC has been demonstrated in several conflicting studies [127,128]. Exosomal miR-25-3p is involved in VEGF signaling pathway activation and enhanced vascular permeability through suppression of *KLF2* and *KLF4* (Krüppel-like factor) in HUVECs [39]. The KLF2/HIF-1α/Notch-1 signaling axis suppresses CRC proliferation and activates apoptosis [129]. KLF4 has a tumor suppressor function and low expression is associated with poor survival of CRC patients [130]. 

MiR-181a-5p: miRNA-profiling analysis revealed significant overexpression of miR-181a in liver metastatic compared with non-metastatic CRC. Moreover, in vitro studies have shown that lenti-miR-181a targets Wnt inhibitory factor-1 (*WIF-1*) and can boost tumor progression, metastasis, and EMT [40]. MiR-181a-5p promotes angiogenesis through SRC/VEGF signaling. *SRC1N1* as a direct target of miR-181a-5p inhibits SRC and subsequently suppresses the VEGF pathway [40]. However, in contradiction to its angiogenic role, miR-181a-5p was also shown to suppress *MMP-14* and reduce angiogenesis in CRC [131]. Several studies have confirmed an angiogenic function of MMP-14. It mediated corneal angiogenesis through *VEGFR1* cleavage and was upregulated in proliferative diabetic retinopathy [132,133]. Another report showed that MMP-14 promotes invasiveness and angiogenesis through VEGF and PTTG [134]. Decreased expression of miR-181a after anti-EGFR treatment in CRC suggests an angiogenic activity of this miRNA.

MiR-194: miR-194 contribution to carcinogenesis has been studied in various types of cancer but its role in CRC remains controversial. MiR-194 is involved in EMT induction and invasion in CRC cell lines [135]; nevertheless, its downregulation in CRC tissue compared to adjacent non-cancerous tissue and its association with inhibition of cell proliferation via regulation of the MAP4K4/c-Jun/MDM2 signaling pathway indicate tumor suppressive features of miR-194 [136]. It has been reported that the miR-194 promoter has a binding site for Snail which leads to miR-194 downregulation and *THBS1* upregulation in snail-mediated EMT in HT-29 cells [137]. THBS1 (thrombospondin 1) is an intrinsic inhibitor of angiogenesis which also has a suppressive effect on CRC proliferation and migration. It can be regulated post-transcriptionally by miR-194 in addition to being induced by p53 suggesting that the P53/THBS1axis is regulated by miR-194 in CRC [41]. 

MiR-27a: miR-27a promotes CRC proliferation, migration, and invasion by downregulation of *RXRα* [138]. RXRα interacts with β-catenin and suppresses the Wnt/β-catenin signaling pathway [139]. Immunogenic cell death induced by chemotherapeutic drugs such as mitoxantrone and oxaliplatin can be impaired by miR-27a overexpression in CRC. MiR-27a directly targets *calreticulin*, a mediator for eliciting immunogenic cell death [140]. Conditioned media from high miR-27a expressing CRC cell lines promote angiogenesis in HUVECs; moreover, the angiogenic potential of miR-27a has been demonstrated in a mouse xenograft model of CRC [141]. Increased miR-27a expression in human lymphatic endothelial cells (HLECs) stimulates lymphangiogenesis under co-culture with colon cancer cells [43].

MiR-130b: miR-130b expression is notably elevated in CRC and leads to poor prognosis in a PPARγ-dependent manner [42]. However, miR-130b may also have a tumor suppressor function in CRC by attenuating migration and invasion through targeting *β1-integrin* [142]; hence, the exact role of miR-130b in CRC is still debatable. *PPARγ* suppression by miR-130b contributes to increased VEGF expression, suggesting a pro-angiogenic function of miR-130b in CRC [42]. 

## 5. Anti-Angiogenic MiRNAs in CRC

MiR-27b: miR-27b expression is downregulated in CRC tissue and plays tumor-suppressive role through *Rab3D* inhibition [143]. *VEGFC* suppression by miR-27b demonstrates antiangiogenic function of miR-27b in CRC [33]. VEGFC is one of the VEGF isoforms that regulates lymphangiogenesis [144]. VEGFC activates p38MAPK and NOTCH1 which leads to angiogenesis [145]. VEGFC upregulation is involved in CRC immune evasion and tumor growth [146,147].

MiR-206: Prognostic value of miR-206 has been shown in CRC patients. Low expression of miR-206 in cancerous tissue is linked to poor overall survival of patients [148] The anti-tumor effect of miR-206 in CRC is mediated via targeting various genes such as transmembrane 4 L6 family member 1 (*TM4SF1*) and *VEGF* [149]. NOTCH3 signaling suppression by miR-206 is associated with apoptosis induction, reduced migration and metastasis in CRC [150]. Furthermore, low expression of miR-206 resulting in higher BCL-2 expression increased 5-fluorouracil (5-FU)-resistance in colon cancer [151]. It has been demonstrated that CCL19 can inhibit angiogenesis in CRC by miR-206 upregulation which subsequently leads to negative regulation of the Met/ERK/Elk-1/HIF-1α/VEGF-A pathway [44].

MiR-126: Reduced expression of miR-126 has been observed in metastatic CRC and correlated with poor clinical outcome [152]. MiR-126 suppresses colon cancer migration, invasion, and proliferation via targeting various targets such as *CXCR4*, *IRS1*, *SLC75A*, and *TOM1* [153,154]. Multiple signaling pathways are negatively regulated by miR-126 including RhoA/ROCK, AKT, and ERK1/2 [155,156,157]. Epigenetic suppression of miR-126 leads to VEGF-mediated angiogenesis [45]. However, Hansen et al. have indicated that miR-126 overexpression is correlated with high level expression of VEGFR-2 and consequently CRC neo-angiogenesis [158]. Circulating miR-126 could be a predictive biomarker in metastatic CRC for treatment with the monoclonal anti-VEGF antibody Bevacizumab since [159] poor patient response to Bevacizumab was associated with increased levels of extracellular miR-126 in plasma.

MiR-143: Increased circulating serum miR-143 is a predictive biomarker for favorable neo-adjuvant therapy response in advanced rectal cancer [160]. MiR-143 downregulation is correlated with poor prognosis and promotes oxaliplatin-based chemotherapy response through downregulation of (insulin-like growth factor-I receptor) *IGR-IR* and superoxide dismutase 1 (*SOD1*) [46,161]. The role of IGR-IR in angiogenesis is in association with PI3K/AKT. Therefore, the PI3K/AKT/HIF-1/VEGF pathway is a possible target in miR-143 anti-angiogenic function [46].

MiR-1249: The miR-1249 promoter has a p53 binding site and its expression is induced by P53 [47]. Chen et al. have shown elevated expression of miR-1249 is associated with decreased CRC cell metastasis and angiogenesis by blocking of *VEGFA* and high mobility group AT-hook 2 (*HMGA2*) [47]; Moreover, by in vivo angiogenesis assays, they have demonstrated that an inverse correlation between MiR-1249 and *CD31* which is linked to anti-angiogenic functions of miR-1249. The EC marker CD31 participates in the intercellular junction of ECs [162]. 

MiR-590-5p: Zhang, G.-J. et al. have reported that miR-590 upregulation predicts poor prognosis for CRC patients [163]. In addition, they have shown that *PTEN* expression is suppressed by miR-590 in CRC suggesting an oncogenic role of this miRNA via the PTEN/PI3K/Akt/mTOR pathway. Hypoxia-regulated miR-590-5p dysregulation in CRC is associated with tumor metastasis by depleting *RECK* (reversion-inducing cysteine-rich protein with Kazal motifs) levels [164]. RECK regulates angiogenesis through MMP modulation [165]. MiR-590-5p is involved in VEGF- dependent angiogenesis by NF90 (targeting nuclear factor 90) suppression [48]. NF90 promotes angiogenesis by induction of HIF-1α and VEGF expression through the PI3K/AKT signaling pathway [166].

MiR-218: Several studies have shown downregulation and anti-tumoral functions of miR-218 in CRC samples and cell lines. MiR-218 negatively regulates EMT and angiogenesis. Silencing the expression of *CTGF*, *VEGFA* and *ANGPT2* after miR-218 transfection into CRC cell lines verified the anti-angiogenic role of miR-218 [49]. The angiogenic potential of connective tissue growth factor (CTGF) and ANGPT2 in association with miRNAs has been shown in various studies. MiR-210 upregulation by CTGF mediates VEGF expression in osteoarthritis [167]. CTGF decreased miR-543 and leads to angiogenesis by ANGPT2 upregulation in osteosarcoma [168].

MiR-6868-5p: Decreased expression of miR-6868-5p is associated with tumor-mediated angiogenesis in CRC patients [50]. MiR-6868-5p suppresses forkhead box M1 (*FOXM1*) which is an oncogene and angiogenesis mediator. Moreover, there is a negative feedback loop between miR-6868-5p expression and *FOXM1*. MiR-6868-5p inhibits IL-8 indirectly through *FOXM1* suppression [50]. FOXM1 has been demonstrated to crosstalk with the TGF-β and Wnt pathways and its expression is correlated with MMP-2, MMP-9, VEGF, and urokinase-type plasminogen activator (uPA), and hence, FOXM1 can modulate angiogenesis, migration, and proliferation of tumor cells [169,170]. 

MiR-107: Studies on miR-107 functions in CRC show conflicting results. Transferrin receptor 1 (*TFR1*) is a direct target of miR-107 and its overexpression promotes proliferation and invasion of CRC cell lines [171]. Although, miR-107 expression is regulated by p53 in CRC, this regulation is not in accordance with tumor suppressive roles of miR-107 [172]. The miR-103/107 family sustains stemness of CRC cells by hyperactivation of Wnt/β-catenin signaling [173]. The predictive value of miR-107 has been demonstrated in metastatic CRC patients in response to chemotherapy [174]. An inverse correlation between miR-107 and hypoxia-inducible factor-1β *(HIF-1β*) suggests an anti-angiogenic function of miR-107 in CRC [51]. *HIF-1β* suppression by p53-responsive miR-107 impairs hypoxic signaling and angiogenesis in CRC [51].

MiR-526b-3p: Although angiogenic potential of miR-526b-3p has not been investigated in CRC but it negatively regulates *HIF-1α* therefore, this reverse relationship might be involved in angiogenesis [175,176]. We have performed a bioinformatics analysis by DIANA-miRPath v3 [177] for finding predicted angiogenesis-related signaling pathways that could be regulated by miR-526b-3p. This suggested that miR-526b-3p blocks targets such as *VEGFA* and *PTEN* in the MAPK and mTOR signaling pathways (data not shown). 

MiR-150-5p: Reports regarding miR-150-5p function in CRC show contradictory results. Decreased expression of miR-150-5p was proposed as a predictive biomarker for poor adjuvant chemotherapy response in CRC patients [178]. Moreover, low serum levels of exosomal miR-150-5p and miR-99b-5p can distinguish CRC patients from healthy individuals [179]. Despite these reports, Liu and Wang et al. have introduced miR-150-5p as an oncomiR in CRC progression [180]. They have shown that *p53* is targeted by miR-150-5p in CRC. MiR-150-5p expression was stimulated by the Wnt/β-catenin signaling pathway and, furthermore, miR-150-5p could repress cAMP response element-binding protein *(CREB)* expression which causes EMT in colon cancer [181]. Several studies have shown long non-coding RNA (lncRNA) and miRNA interactions in cancer development. ZNFX1 antisense RNA1 (ZFAS1) is a lncRNA which acts as miRNA sponge and can indirectly regulate miR-150 targets (*ZFAS1* and *VEGFA*) [52]. Indeed, the angioinhibitory function of miR-150-5p is mediated by *VEGFA* suppression which subsequently leads to tumor growth inhibition in CRC. 

MiR-125a-3p & 5p: Upregulation of exosomal miR-125a-3p in CRC patients’ plasma can be a useful biomarker in early detection of CRC [182]. MiR-125a-3p inhibits angiogenesis in CRC by targeting fucosyltransferases (*FUT5* and *FUT6*); FUT contributes to CRC progression via the PI3K/Akt signaling pathway [53]. FUTs are endoplasmic reticulum and Golgi resident membrane-bound proteins which regulate several signaling factors such as VEGFR and the PI3K/AKT signaling pathway [183]. Serum fucosylation profiles could be valuable biomarkers in different types of cancer [183]. MiR-125a-5p tumor suppressive functions in CRC have been demonstrated in several studies. *TAZ* is a direct target of miR-125a-5p and its overexpression reverses CRC cell invasion and migration mediated by miR-125a-5p [184]. Apoptosis induction via targeting of *BCL2*, *BCL2L12*, and *Mcl-1* in colon cancer cells by miR-125a-5p is another aspect of the tumor suppressive role of this miRNA [185]. MiR-125a-5p also suppresses tube formation of HUVECs by targeting *VEGFA* [54].

MiR-140-5p: MiR-140-5p is involved in EMT activation and maintenance of cancer stem cell characteristics in CRC through *SMAD2* downregulation downstream of TGFβ signaling [186]. MiR-140-5p and lncRNA cancer susceptibility 19 (CASC19) interaction can abolish the oncogenic role of CASC19 in CRC [187]. MiR-140-5p downregulation in CRC tissue correlated with TNM stage and poor overall survival of CRC patients [188,189]. Zhang et al. have shown *VEGFA* as a valid direct target of miR-140-5p in CRC; however, tube formation ability was not evaluated in this study [55].

MiR-145: MiR-145 is recognized as a tumor-suppressive miRNA in CRC. MiR-145 inhibits colon cancer cell migration and invasion through post-transcriptional suppression of p21-activated kinase 4 (*PAK4*) [190]. PAK4 acts at the cross-point between MAPK and Wnt/β-catenin signaling and is essential for cell migratory ability and survival [191]. Interaction between oncogenic colon cancer-associated transcript-2 (*CCAT2*) lncRNA and miR-145 leads to decreased maturation of pre-miR-145 [192]. Glioma-derived exosomal CCAT2 transfer to ECs and induction of VEGF expression has been reported [193]. The 70kDa ribosomal S6 kinase (*P70S6K1*) is a direct target of miR-145 and exerts its pro-angiogenic function through VEGF and HIF-1α expression [56,194]. Another study showed a contradictory role of miR-145 in angiogenic response. CRC–derived exosomes containing miR-145 could transfer information to TAMs and induce M2 polarization of TAMs which overexpress VEGF and participate in tumor angiogenesis [195].

MiR-195-5p: MiR-195-5p is downregulated in CRC tissue and is associated with chemotherapy sensitivity in CRC by targeting glycerophosphodiester phosphodiesterase domain containing 5 (*GDPD5*) repression [196,197]. Downregulation of *WNT3A* by miR-195-5p inhibits CRC cell migration and proliferation [198]. WNT3A overexpression exerts its pro-angiogenic function in CRC cell lines by VEGFR2 and VE-cadherin upregulation [199]. DKK1 is a Wnt/β-catenin antagonist that can repress WNT3A activity and serves as tumor-suppressive factor in CRC [200]. Another study showed that miR-195-5p downregulates several angiogenic genes such as *VEGFA*, *DLL4*, *ENG*, *HIF-1α*, and *HIF-1β* in CRC [57]. 

MiR-622: MiR-622 is downregulated in CRC and suppresses migration, invasion, and proliferation of cancer cells by targeting various targets such as dual-specificity tyrosine phosphorylation-regulated kinase 2 (*DYRK2*) and Kirsten rat sarcoma (*K-Ras*) [201]. MiR-622 expression can be induced by radiotherapy and causes radioresistance through retinoblastoma protein (*Rb*) inhibition in CRC [202]. Fang et al. have reported that miR-622 negatively regulates angiogenesis both in vitro and in vivo by (C-X-C chemokine receptor 4) *CXCR4* and *VEGF* suppression [58]. HUVEC treatment with CRC cell line-derived conditioned medium suppresses tube formation and migration by CXCR4 inhibition; moreover, CXCR4 is upregulated due to hypoxia in CRC and CXCR4/VEGF/HIF1α signaling has a correlation with the TNM stage [203,204].

MiR-452: MiR-452 activates Wnt/β-catenin through *GSK3β* suppression and might evoke cell cycle progression in CRC by blocking cyclin-dependent kinase inhibitor 1B (CDKN1B) [205]. Mo et al. have demonstrated that miR-452 targets VEGF and inhibits tumor angiogenesis [59]. Furthermore, they have shown that miR-452-mediated suppression of *VEGF* resulted in SRC/BRAF/MAPK signaling pathway inhibition. 

## 6. Conclusions

Angiogenesis is a key process in physiological and pathological conditions and is controlled by various angiogenic and anti-angiogenic factors. The imbalance between these factors leads to dysregulation of angiogenesis during development of tumors including CRC. MiRNAs are important mediators in this context and, consequently, have emerged as novel diagnostic, prognostic, and predictive biomarkers in CRC. MiRNAs can target angiogenesis-related oncogenic or tumor suppressor mRNAs in different signaling pathways in either tumor cells or cells of the TME and thereby exert angiogenic or anti-angiogenic functions. Moreover, exosomal miRNAs can shuttle between CRC cells and the TME and transduce angiogenesis-regulating signals between different cellular components to either boost or impair tumor progression. Altogether, understanding the multiple roles of miRNAs in CRC angiogenesis could help to identify new biomarkers and improve miRNA-based antiangiogenic therapies.

## Figures and Tables

**Table 1:** MiRNAs involved in regulation of angiogenesis during wound healing.

MiRNA	Functions	Ref.
MiR-21	TGF-β–mediated wound contraction Exosomal miR-21 promotes angiogenesis through PTEN and SPRY1 suppression	[16,17]
MiR-26a	Impairs angiogenesis by targeting SMAD1	[18]
MiR-27b	Facilitates angiogenesis by downregulation of THBS1, Sema6A and p66Shc	[19]
MiR-92a	Angiogenesis in fracture healing could increase by miR-92a inhibition	[20]
MiR-125a	Enhances angiogenesis by DLL4 repression	[21]
MiR-126	Blood vessel formation through VEGF and bFGF signaling	[22]
MiR-135a-3p	Inhibits angiogenesis by targeting of VEGF-HIP1-p38K signaling	[23]
MiR-148b	Promotes wound healing by TGFβ signaling regulation	[24]
MiR-199a-5p	Suppresses angiogenesis by targeting of the Ets-1-MMP1 pathway	[25]
MiR-615-5p	Suppresses angiogenesis by regulation of the VEGF-AKT/eNOS signaling pathway	[26]

**Table 2:** Angioregulatory miRNAs in CRC.

Angiogenic MiRNAs	Target Genes or Molecular Pathways Involved in Angiogenesis	Ref.
MiR-92a	DKK3 and claudin-11	[36]
MiR-1246	PML inhibition and SMAD 1/5/8 signaling activation	[37]
MiR-1229	HIPK2	[38]
MiR-25-3p	KLF2 and KLF4	[39]
MiR-181a-5p	SRC/VEGF signaling	[40]
MiR-194	THBS1	[41]
MiR-130b	PPARγ	[42]
MiR-27a	SMAD4	[43]
**Antiangiogenic MiRNAs**		
MiR-27b	VEGFC	[33]
MiR-206	Met/ERK/Elk-1/HIF-1α/VEGF-A pathway inhibition	[44]
MiR-126	VEGFA	[45]
MiR-143	PI3K/AKT/HIF-1/VEGF	[46]
MiR-1249	VEGFA and HMGA2	[47]
MiR-590-5p	NF90	[48]
MiR-218	CTGF, VEGFA and ANGPT2	[49]
MiR-6868-5p	FOXM1	[50]
MiR-107	HIF-1β	[51]
MiR-150-5p	ZFAS1 and VEGFA	[52]
MiR-125a-3p & 5p	FUT5 and FUT6/ VEGFA	[53,54]
MiR-140-5p	VEGFA	[55]
MiR-145	P70S6K1	[56]
MiR-195-5p	VEGFA, DLL4, ENG, HIF-1α and HIF-1β	[57]
MiR-622	CXCR4 and VEGF	[58]
MiR-452	SRC/BRAF/MAPK signaling pathway inhibition	[59]

**Table 3:** CRC related angio-miRNAs in other types of cancer and/or disorders (excluding CRC).

MiRNAs	Cancer and/or Other Disorders	Angiogenic/Anti-Angiogenic	Target Genes or Molecular Pathways Involved in Angiogenesis	Ref.
MiR-92a	1. Vascular injury 2. Mouse hind-limb ischemia model	Antiangiogenic	1. JNK and ERK1/2 pathway is activated following by miR-92a suppression. 2. Integrin subunit a5 (ITGA5)	[60,61]
MiR-1246	Corneal neovascularization	Antiangiogenic	Angiotensin-converting enzyme (ACE)	[62]
MiR-181a-5p	Chondrosarcoma	Angiogenic	RGS16 (CXC chemokine receptor 4 (CXCR4) signaling)	[63]
MiR-27a/b	-	Angiogenic	SEMA6A	[64]
MiR-206	1. Breast cancer 2. Non-small cell lung cancer 3. Laryngeal cancer	Antiangiogenic	1. VEGF, MAPK3 and SOX9 2. c-Met/PI3K/AKT/mTOR pathway 14-3-3ζ/STAT3/HIF-1α/VEGF signaling 3. VEGF	[65,66,67,68]
MiR-126	1. Gastric cancer 2. Oral cancer 3. Breast cancer 4. Spinal cord injury 5. Ischemic mouse brain 6. Arteriosclerosis	Antiangiogenic	1 & 2: VEGF-A 3. VEGF/PI3K/AKT signaling pathway 4. SPRED1, PIK3R2 & VCAM1 5. PTPN9 suppression and AKT and ERK signaling pathways activation 6. PI3K/AKT JAK2/STAT5 signaling pathway suppression following by curcumin treatment	[69,70,71,72,73,74,75]
MiR-140-5p	1. Breast cancer 2. Glioma 3. Larynx carcinoma 4. mouse model of Retinopathy	Antiangiogenic	1. VEGF 2. VEGFA/MMP 2 signaling 3. VEGFA 4. TMOD3	[76,77,78,79]
MiR-143/miR-145	Lung cancer	Antiangiogenic	Camk1d	[80]
MiR-145	1. Breast cancer 2. Neuroblastoma	Antiangiogenic	1. VEGF and N-RAS 2. HIF-2α	[81,82]
MiR-590-5p	Oral squamous cell carcinoma	Angiogenic	CD44 and VE-cadherin	[83]
MiR-107	1. Glioma 2. Liver cancer 3.Ischemia-induced cerebral injury	1 & 2. Antiangiogenic 3. Angiogenic	1. VEGF 2. HULC mediated E2F1–SPHK1 signaling 3. Dicer-1	[84,85,86]
MiR-526b	Breast Cancer	Angiogenic	NFKB pathway PI3K/Akt signaling	[87]
MiR-150-5p	1. Paclitaxel-resistant ovarian cancer 2. Rheumatoid arthritis	Antiangiogenic	1. Notch3 signaling 2. MMP14 and VEGF	[88,89]
MiR-125a-3p	1. Renal cancer 2. Gastric cancer 3. Hepatocellular carcinoma	Antiangiogenic	1 & 2. VEGF 3. VEGF and MMP13 (however angiogenesis assay was not performed in this study)	[90,91]
MiR-125b-5p	1. Ovarian cancer 2. Hepatocellular carcinoma	Antiangiogenic	1. EIF4EBP1 2. PIGF	[92,93]
MiR-195-5p	1. Squamous cell lung cancer 2. Ovarian cancer 3. Hepatocellular carcinoma 4. Prostate cancer	Antiangiogenic	1. VEGF 2. PSAT1-dependent GSK3β/β-catenin signaling pathway 3. VEGF, VAV2, and CDC42 4. PRR11	[94,95,96,97]
MiR-218	1. Gastric cancer 2. Prostate cancer	Angiogenic	1. ROBO1 2. RICTOR	[98,99]
MiR-452	Breast cancer	Antiangiogenic	SNAI2	[100]
